# Cassava Starch Films with Anthocyanins and Betalains from Agroindustrial by-Products: Their Use for Intelligent Label Development

**DOI:** 10.3390/foods11213361

**Published:** 2022-10-26

**Authors:** Carlos M. Otálora González, Laura I. Schelegueda, Víctor M. Pizones Ruiz-Henestrosa, Carmen A. Campos, María F. Basanta, Lía N. Gerschenson

**Affiliations:** 1 Consejo Nacional de Investigaciones Científicas y Técnicas (CONICET), Departamento de Industrias, Facultad de Ciencias Exactas y Naturales, Universidad de Buenos Aires (UBA), Argentina; 2 Instituto de Tecnología de Alimentos y Procesos Químicos (ITAPROQ), UBA-CONICET, Ciudad Universitaria, Avenida Intendente Guiraldes 2620, Ciudad Autónoma de Buenos Aires 1428, Argentina

**Keywords:** films, starch, plant by-products, natural pigments, fish spoilage detection

## Abstract

The development of biodegradable packaging materials has become a widely addressed topic in recent years. Microparticles generated from *Brassica oleracea* var. *capitata* f. *rubra* (red cabbage, RC) and *Beta vulgaris* L. var. conditiva (beetroot, BR) which contained anthocyanins or betalains, were included in the formulation of edible films based on cassava starch (CS) giving origin to films CSRC, CSBR, or CSBC (mixture of both particles). The inclusion of the filler determined an increase in the stress at rupture from 0.8 MPa (CS) to 1.2 MPa (CSRC) or 1.0 MPa (CSBC), of the contact angle from 2.6° to 13.8° (CSBR) or 19.6° (CSBC). The use of these films for developing a smart label for hake packaging and the study of the TBV-N content, the microbiological characteristics of the muscle, and the color changes of the label with time, allowed us to conclude that the films CSRC and CSBC would be suitable for sensing the deterioration of packaged and chilled hake and that the color change of the label CSBC was completely consistent with fish muscle deterioration. As the microparticles can be obtained from by-products of the production and industrialization of plant tissues, the composite films and the smart labels developed can contribute not only to the development of safe food but also to the addition of value to those residues and to environmental protection.

## 1. Introduction

Packaging based on non-biodegradable synthetic polymers has caused serious environmental problems [1]. Therefore, the development of biodegradable packaging materials has become a widely addressed topic in recent years. Among the biodegradable materials are edible films that can support antimicrobials and antioxidants [2,3] that confer bioactivity useful for improving food quality. Edible films based on polysaccharides or proteins have lower mechanical resistance and higher permeability to water vapor than packaging materials based on petroleum-derived materials, and the addition of fillers has been tested to improve these characteristics [4]. Starch, an abundant filmogenic biopolymer with numerous industrial applications [5], has been extensively used for edible film development [6].

Intelligent packaging monitors the quality of food and provides information about its status contributing to food safety. It must be non-toxic and sensitive to deterioration factors [7]. Synthetic pigments such as bromocresol, methyl red, and xylenol blue, potentially toxic and carcinogenic, are often used in the development of these packages when a color change is the factor that allows the evaluation of quality by the consumer [8]. For this reason, research has been carried out focused on its replacement by natural pigments which can be obtained from edible plant tissues, either in the form of liquid extracts or powders [9,10,11,12].

Different authors reported that films containing natural pigments can be used as intelligent packaging to monitor the freshness of food products. For this purpose, Qin et al. [11] and Jamróz et al. [13] informed the use of labels with betalains and constituted by furcellaran or starch/polyvinyl alcohol (PVA), respectively. Liang et al. [14], Zhang et al. [15], and Cheng et al. [16] reported the use of labels with anthocyanins and based on Artemisia gum or starch/PVA or starch, respectively.

FAO [17] proposes the reduction in waste and the minimization of losses in food production to contribute to greater efficiency and the better distribution of food, which would help end hunger for millions of people. Residues and their disposal represent important costs for industries, often inadequately evaluated. These facts remark the existence of two parallel problems: waste management and reduction in resources, which can be solved simultaneously through the use of these residues as an alternative source of carbon to produce chemical compounds and other commercially viable products [18]. Plant tissues originated as by-products of agroindustrial processes and can be used as a source of polysaccharides, antioxidants, and pigments, adding value to raw materials while contributing to sustainable development and environmental protection. In particular, natural pigments like betalains and anthocyanins can be obtained from the by-products of beetroot and red cabbage production and industrialization [19,20].

Fishery products are highly susceptible to deterioration due to their high moisture and non-protein nitrogen content and pH close to 7 [13,21]. The capture of the fish determines the beginning of the deterioration and produces a series of biochemical and microbiological reactions that lead to the loss of freshness. Degradation of protein and non-protein nitrogen due to endogenous enzymes and microbiological activity gives origin to the formation of dimethylamine, trimethylamine, formaldehyde, and ammonia, which constitute total volatile basic nitrogen (TVB-N). The evaluation of the latter is used as an index of freshness since it shows a good correlation with microbiological growth [22]. In accordance with the regulations of the European Economic Community, it is necessary for the fish to have a maximum TVB-N content of 30 mg/100 g in order to be marketed [23].

The performances of anthocyanins and betalains from different sources have been explored for spoilage detection. As ammonia produces different color changes in these pigments, they can be used to monitor the quality of protein foods [14,24]. For example, Qin et al. [11] informed that starch-based films containing red pitaya betalains could detect spoilage in shrimp. However, according to our knowledge, the incorporation into cassava starch films of a mixture of red cabbage (RC) and beetroot (BR) microparticles as conveyors of anthocyanins and betalains has not been explored in the literature in relation to the effect of that incorporation into films’ physicochemical, mechanical, and structural properties and on the capacity for detecting fish spoilage. Otálora et al. [19] informed that the low water activity of the BR powder and its lignin content ensured effective protection of the pigments from thermal degradation, making these microparticles an adequate conveyor and stabilizer of betalains.

In this study, our aim was: (1) to produce composite films based on cassava starch with the incorporation of microparticles that can be obtained from *Brassica oleracea* and/or *Beta vulgaris* tissues and to characterize their water solubility, wettability, mechanical properties, and structural properties; (2) to evaluate the potential use of the films as smart labels for monitoring fish spoilage; (3) to analyze the potential benefits of using mixtures of anthocyanins and betalains to obtain more precise indications of fish spoilage.

The development of these films will contribute to the safety and sustainability of agroindustrial processes as well as to the health of the consumer.

## 2. Materials and Methods

### 2.1. Chemicals

The food-grade native cassava starch (amylose content: 24 g 100 g^−1^; amylopectin content: 76 g 100 g^−1^) was obtained from Bernesa S.A. (Lomas de Zamora, Buenos Aires, Argentina). Glycerol (Biopack, Buenos Aires, Argentina) and potassium sorbate (Sigma, Saint Louis, MO, USA) used were of analytical grade. Deionized water (Milli-QTM, Manassas, VA, USA) was used. The other chemicals of analytical grade were from Merck Química (Buenos Aires, Argentina).

### 2.2. Red Cabbage and Beetroot Powder Obtention

Powders were obtained according to Otálora et al. [19,20]. Briefly, RC and BR tissues were washed, cut into slices, and blanched. The samples were freeze-dried, milled, and sieved using a vibratory shaker to obtain powders with a microparticle size smaller than 105 μm. The total contents of betalains and anthocyanins in BR and RC microparticles were 0.73 mg betalains/g and 3.68 mg cyanidin-3-glucoside (C3G)/g, respectively [20].

### 2.3. Preparation of the Intelligent Films

Control films were prepared by casting according to Otálora et al. [20]. Cassava starch, glycerol, potassium sorbate (5 g:2 g:0.2 g), and distilled water up to 100 g were mixed (300 rpm) under heating (rate: 2 °C/min) up to 82 °C (control film, CS). Intelligent films were prepared by adding to the control formulation 1.5% *w*/*w* of different powders, reducing the quantity of water. Films obtained were constituted by cassava starch and BR (CSBR film), cassava starch and RC (CSRC film), and cassava starch and a mixture of BR (0.3% *w*/*w*) and RC (1.2% *w*/*w*) (CSBC film). The choice of powder concentration was based on previous tests (data not shown) that allowed the selection of formulations that gave origin to self-supported, flexible films. The powder was included in the formulation and hydrated with a fraction of the total water (powder/water ratio, 1/10), after mixing the rest of the components at 82 °C; this incorporation was performed with stirring at 300 rpm for 1 min. Each film-forming solution (24 g) was cast into silicone molds (diameter = 7 cm) (Silikomart, Venice, Italy) and dried in a controlled temperature chamber at 45 °C (FAC, Buenos Aires, Argentina) for 20 h. The films were equilibrated to a water activity of 0.575 (25 °C) previous to their characterization.

### 2.4. Characterization of the Films

#### 2.4.1. Determination of Betalain and Anthocyanin CONTENT in the films

An amount of 1 g of edible film was treated with Milli-QTM water and the supernatant aqueous extract was used for measurement of the pigment contents [20].

#### 2.4.2. Thickness and Water Solubility

These characteristics were determined according to Otálora et al. [25].

#### 2.4.3. Color

Color parameters were determined with a Minolta spectrophotometer (Minolta CM-600D, Tokyo, Japan), according to Otálora et al. [20]. The change in color (∆E*) of the films was calculated in relation to color attributes of control films.

#### 2.4.4. Mechanical Properties

Mechanical properties were evaluated using a universal Instron testing machine model 3345 (Instron Corp., Norwood, MA, USA). Force applied (F) and sample changing length (L) were registered along the assay. The stress at rupture (SR), σ, and the deformation at rupture (DR), ε, were calculated.

#### 2.4.5. Contact Angle and Wettability

The contact angle and wettability were determined according to Otálora et al. [25].

#### 2.4.6. Scanning Electron Microscopy (SEM)

Film microstructure was characterized by means of a ZE122 SEM Supra 40 scanning electron microscope (Carl Zeiss, Jena, Germany). The cross sections and the surface of films were metalized with a platinum layer prior to observation. The image analysis was performed by means of SmartSEM^®^ V05.06 software (Carl Zeiss, Jena, Germany).

#### 2.4.7. Fourier Transform Infrared Spectroscopy (FTIR)

The IR spectra of films were recorded with a Nicolet iS50 FT-IR (Thermo Scientific Nicolet, Waltham, MA, USA) spectrometer using diamond-attenuated total reflectance (ATR) technique with a 45° incident angle. The spectrum data was recorded in the range of 500–4000 cm^−1^ wavenumber at a resolution of 4 cm^−1^ with OMNIC software version 7.3 (Thermo Electron Corp., Waltham, MA, USA).

### 2.5. Application of the Films as Intelligent Labels to Detect Fish Spoilage

Fresh Argentine hake (*Merluccius hubbsi*) was purchased in the local market and transported to the laboratory preserved on ice. The specimens were washed under tap water, eviscerated, and filleted in sterile conditions. Fillets were washed with sterile chlorinated water (7 mg/L), cut into 20 g pieces, and air-packed in PET trays (height 3 cm and diameter 6 cm) with transparent covers, leaving a 1 cm headspace between the fish surface and the lids. Previously, the PET trays and their lids were sterilized by placing them under laminar flow and at a distance of 65 cm from a UV-C light (20 W, 60 cm length) for 1 h. The films CSRC, CSBR, and CSBC were cut into 4 × 4 cm square pieces which were fixed to the container lids by means of a double-sided tape that was dispensed in the corners, with the purpose of acting as smart labels.

The preparation of the systems was performed under laminar flow and in triplicate. They were stored at 4 °C and independent samples were withdrawn at selected times to conduct fish microbiological and physicochemical analysis until smart labels change their color.

#### 2.5.1. Microbiological Analyses

Total mesophilic aerobic bacteria (TMAB) were enumerated in PCA agar (Biokar Díagnostics, Beauvais, France) according to the plate count method described by Schelegueda et al. [21]. Plates were incubated at 30 °C for 48 h. Data were recorded as colony-forming units (CFU/g) and expressed as log10 CFU/g.

#### 2.5.2. Physicochemical Analyses

Total volatile basic nitrogen (TVB-N) content was determined by Conway’s method [16]. Data were expressed as mg of TVB-N/100 g fish.

#### 2.5.3. Color Analysis

The film squares were detached from the cover of the PET package and characterized for their color parameters using the CIELab system and photographic characterization. The total color difference (ΔE*) was calculated using the color data at the start of the trial (zero time).

### 2.6. Statistical Analysis

Non-linear fits were made by means of the Prism 5 utility (GraphPad, San Diego, CA, USA). Statistical analyses of the significance of the differences were carried out using ANOVA with a significance level, α, of 0.05. One-factor (system) ANOVA was performed for the parameters presented in Table 1, and a two-factor (system and time) ANOVA was performed for the parameters presented in Table 2. The Tukey test was used *a posteriori* [26].

## 3. Results and Discussion

### 3.1. Film Characterization

As shown in Table 1, the addition of RC, BR, and BC microparticles to the starch-based films caused an increase in thickness. These could be attributed to an increase in the solids content in the formulation and/or to the volume occupied by the microparticles resulting in the formation of more complex matrices [10,27].

The water solubility (WS) of the films indicates their resistance to an aqueous environment. The incorporation of the microparticles produced significant changes in the WS values for the starch films CSBR and CSBC which can be attributed to the higher sugar content of the powders obtained from beetroot [20], which might have facilitated their solubilization in an aqueous medium [27] and/or to structural changes occurring due to BR powder inclusion in the starch matrix.

The mechanical properties of the films provide information about their strength and deformability, useful for predicting their possible applications. The stress at rupture (SR) is a measure of the film strength, whereas the deformation at rupture (DR) is a measure of the film stretchability. The addition of RC and BC powders (CSBC) or of only RC powders (CSRC) produced an increase in SR and a decrease in DR. Lower DR values were also detected for films with BR powders (CSBR) (Table 1). This is a trend generally reported in the bibliography for composite films and is attributed to the reinforcing of the matrix produced by a filler addition which results in stress increasing and a decrease in extensibility [28]. Luchese et al. [9] obtained similar results, observing a decrease in DR after incorporating blueberry pomace into cassava starch films. Bernhardt et al. [29] constituted composite films based on pectin and observed that the inclusion of cornhusk fiber increased the stress at rupture.

Color is an important property of food packaging films. Table 1 reports the betalain and anthocyanin content and color parameters of the films. Simple films were transparent and with high luminosity and the incorporation of RC, BR, and their mix into the films, produced a decrease in L* and changes in a* and b* according to the pigments present in the microparticles that were incorporated. For example, it is noticeable that CSBR and CSBC showed an increase in red and yellow color as indicated by the increase in a* and b* observed. This trend can be associated with the presence of betaxanthins and betacyanins in BR microparticles [14].

The contact angle is a measure of wettability or surface hydrophobicity [30]. Table 1 shows that CSBR (13.8°) and CSBC (19.6°) showed a higher contact angle than CS (2.6°), indicating that, in those films, the microparticles decreased the wettability and increased the hydrophobicity with respect to CS film, generating an increase in the affinity of the films for more hydrophobic food products like products of animal origin [31]. This trend can be ascribed to the structural changes that might occur due to BR powder inclusion in the starch matrix.

**Figure 1:**
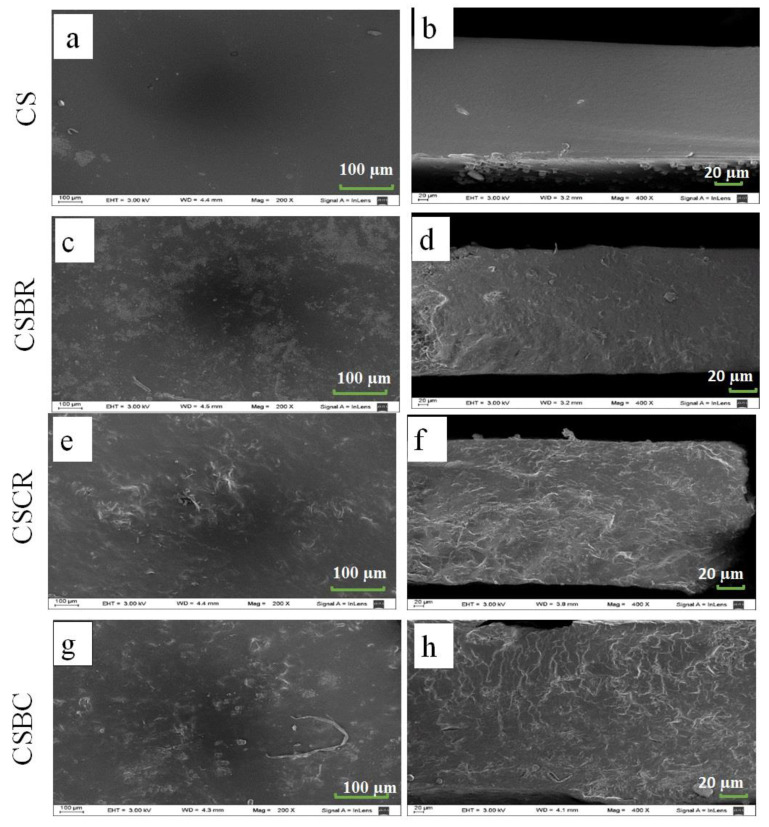
SEM micrographs of the surfaces (**a**,**c**,**e**,**g**) and cross sections (**b**,**d**,**f**,**h**) of the cassava starch film (CS) (**a**,**b**), starch-BR film (CSBR) (**c**,**d**), starch-RC film (CSRC) (**e**,**f**), and starch-BR/RC film (CSBC) (**g,h**).

CS film SEM images (Figure 1a,b) show a homogeneous, smooth, non-porous surface and cross-section, suggesting that starch and glycerol were uniformly mixed in the film [11]. In contrast, composite films showed a heterogeneous, compact, and rough structure that can be observed in the surface and cross sections (Figure 1c–h).

The changes in the structure due to powder inclusion in the starch matrix might have contributed to the increase in stress at rupture reported in Table 1 for CSRC and CSBC.

The structural changes can also explain the increase in contact angle for CSBR and CSBC. The addition of microparticles to the starch matrix determined that the surface of composite films had numerous particles. If the gaps between particles were occupied by air, water droplets probably could not completely fill the gaps and, consequently, the contact angle increased [32].

**Figure 2:**
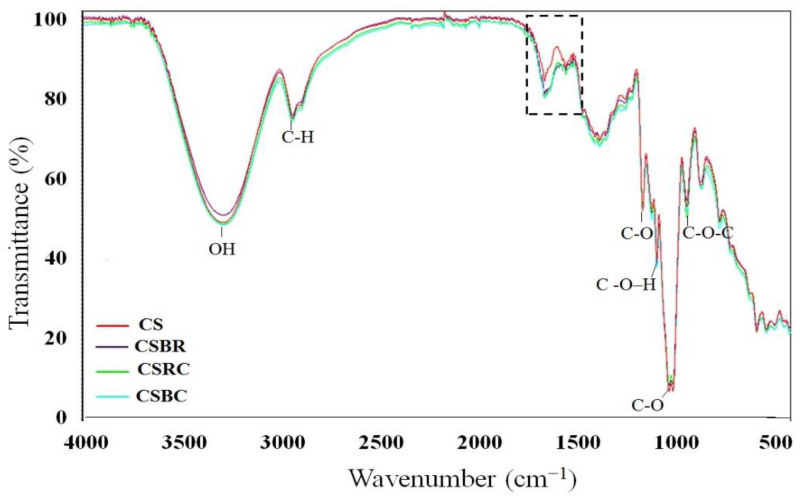
ATR−FTIR of cassava starch film (CS), starch−BR film (CSBR), starch−RC film (CSRC), and starch−BR/RC film (CSBC). The dash line box is signaling the change in the region 1600 to 1700 cm^−1^ due to the addition of the microparticles.

Figure 2 shows the IR spectra of the films. Starch film spectra show the following peaks: 3283 cm^−1^ (O-H stretching), 2938 cm^−1^ (C-H stretching), and 1648 cm^−1^ (O–H bending of bound water). A band at 1150 cm^−1^ corresponds to the C-O deformation and the band at 1078 cm^−1^, to the C-O-H bending. On the other hand, the 926 cm^−1^ band is attributed to the skeletal mode vibrations of the α-1,4-glycosidic, C-O-C bonds. Similar peaks were also reported by Zhai et al. [8], Qin et al. [11], and Zhang et al. [15]. The addition of powders to single films (CS) did not cause the presence of new bands, however, there was a change in the region between 1600 to 1700 cm^−1^, which might be attributed to stretching vibrations of the C=C bonds (1650 cm^−1^) of the aromatic rings of anthocyanins [33] and of the N-H bonds of the amines (1641 cm^−1^) of betalains [34]. This change shows that the powders have been effectively incorporated into the polymeric matrix. The change can also be attributed to changes in the vibration of matrix-bound water (1648 cm^−1^) due to the incorporation of microparticles.

### 3.2. Application of the Films as Intelligent Labels to Detect Fish Spoilage

Microbial spoilage and biochemical reactions are two key reasons for fish freshness loss during storage. The accumulation of metabolites such as amines, aldehydes, ketones, esters, and other low molecular weight substances causes off-flavors conducting sensory product rejection [35]. As mentioned above, the total volatile basic nitrogen (TVB-N) constitutes an essential reference index for evaluating fish freshness [36].

**Figure 3:**
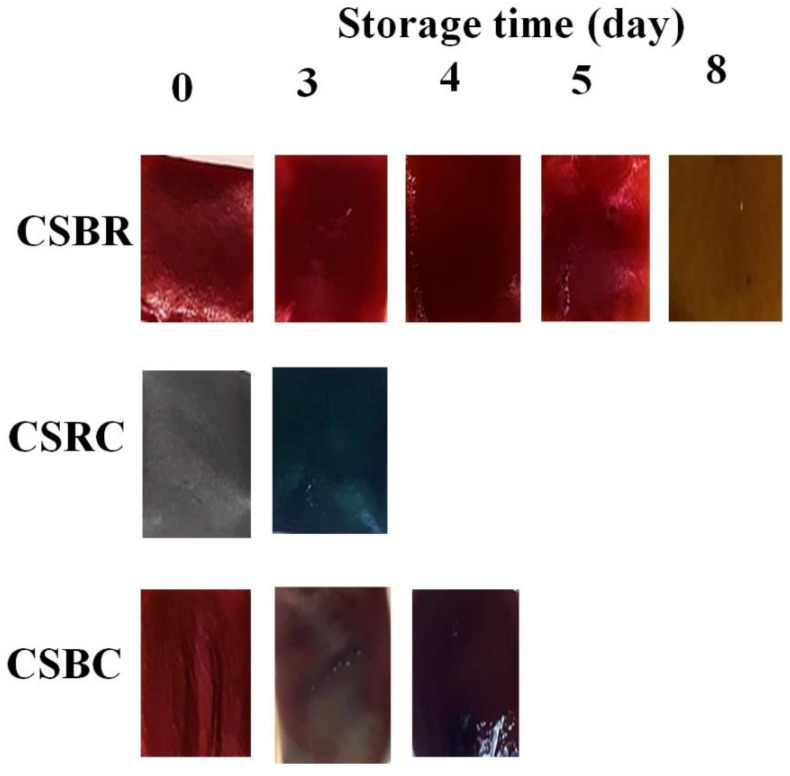
Changes in label color due to fish spoilage at 4 °C.

The ability of the developed films to detect fish spoilage was evaluated by analyzing the microbiological characteristics of fish fillets and TVB-N content, together with color changes occurring in the films. The quality of the raw material was satisfactory, since, in the beginning, TMAB counts were approximately 10^3^ CFU/g, and TVB-N content was less than 6 mg/100 g (Table 2). As expected, the TMAB population and TVB-N content increased significantly during refrigerated storage. Furthermore, given that the only difference between the formulated systems was the composition of the intelligent labels, which does not influence fish spoilage, no differences between systems were observed for the measurements taken at each time (Table 2). After 3 days, both the TMAB count and TVB-N content doubled (Table 2). In this instance, the CSRC films, rich in anthocyanins (RC), turned from gray to blue/green (Figure 3). Due to the fact that the TMAB population was approximately 10^6^ CFU/g and the maximum value allowed in this type of food is 10^7^ CFU/g [8], CSRC films would be useful to indicate to consumers that the end of fish shelf life is close. Four days after baseline, the TMAB count continued increasing, approaching 10^7^ CFU/g. In addition, TVB-N content was at the allowed upper limit. Under these conditions, the CSBC films, rich in anthocyanins/betalains, turned from red to dark purple (Figure 3), warning that the loss of fish freshness has been reached. From the 5th day on, the TMAB population and TVB-N concentration were above the allowed limits (Table 2). However, CSBR films did not show a color change until 8 days of storage. At this point, it was observed that mentioned films, rich in betalains, turned from red to brown (Figure 3). The latter films were not efficient to show the end of fish shelf life, since when a color change occurred, the fillets were in extremely poor hygienic conditions, making their consumption unfeasible.

**Figure 4:**
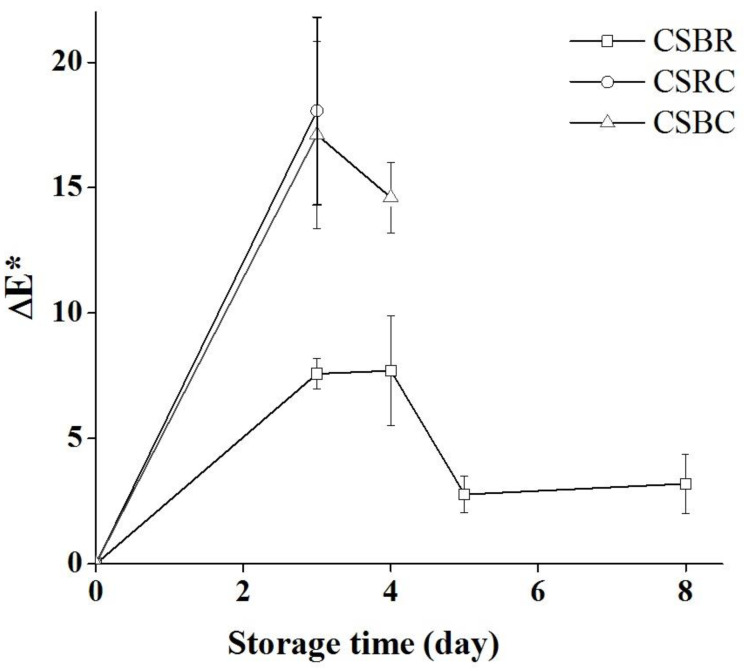
Label color difference (ΔE*) along storage at 4 °C and due to fish spoilage.

The mechanism of action of intelligent labels used in this study to identify hake fillets spoilage is based on their color change due to the effect of the volatile amino compounds formed which diffuse to the headspace [9,37]. It must be noted that in the color analysis carried out on the composite films, ΔE* took values greater than 5 after 3 days (Figure 4) for all of them, but CSBR showed the lowest difference from the input color determining that the change was not perceptible.

Similar results to those obtained in this study have been reported previously. Ezati et al. [38] observed a valid correlation between microbial growth, TVB-N generation, and color changes using alizarin as a colorant supported in a starch–cellulose matrix. Zhai et al. [8] developed starch/polyvinyl alcohol films with roselle anthocyanins and observed significant color changes when refrigerated fish TVB-N content increased from 5 to 20 mg/100 g. In summary, the anthocyanin-containing films (CSRC and CSBC) presented an adequate response to TVB-N, following the specifications of the legislation. It is important to state that in the present research, the smart label is based on an edible film containing natural pigments and that the inclusion of betalains, jointly with anthocyanins, gave origin to a color change (label CSBC) completely consistent with fish muscle deterioration.

The application of the developed films as intelligent labels showed that the films containing anthocyanins (CSRC and CSBC) were adequate for sensing the deterioration of packaged and chilled hake and that the one containing only anthocyanins would be useful for indicating to consumers that the end of fish shelf life is close but the films containing the proposed mix of anthocyanins and betalains warned with more precision the loss of fish freshness. Additionally, the possibility of microparticle obtention from by-products of the production and industrialization of plant tissues constitutes a valuable approach to adding value to raw materials, contributing to sustainable development and environmental protection.

## 4. Conclusions

Composite films based on cassava starch were successfully developed by means of the addition of red cabbage and beetroot microparticles as fillers. These microparticles contained anthocyanins and betalains. In general, the presence of fillers increased the mechanical strength and the hydrophobicity of the films, trends that improved their capacity for acting as packaging films.

The edible composite films with beetroot, red cabbage, or red cabbage/beetroot microparticles, were used to develop smart labels for monitoring Argentine hake spoilage. The labels containing the natural pigments anthocyanins (CSRC) or anthocyanins/betalains (CSBC) showed high sensitivity to total volatile basic nitrogen (TVB-N) content; as a result, they are suitable for sensing the deterioration of packaged and chilled hake. In particular, the inclusion of betalains jointly with anthocyanins (label CSBC) gave origin to a color change completely consistent with fish muscle deterioration and, consequently, this smart label can be efficiently used as a hake spoilage indicator.

As these microparticles can be obtained from the abundant by-products of the production and industrialization of plant tissues, the application of composite films developed as such or as smart labels can contribute not only to ensuring the health of consumers but also to adding value to the raw material and to environmental protection.

The results of the present research contribute to expanding the knowledge in the field of biodegradable packaging materials, edible films, and the use of by-products of plant agroindustrial processes. In particular, the contribution is developed in the following aspects:

- composite films development;
- application of composite films as smart labels;
- obtention of pigments from plant by-products;
- analysis of the potential benefits of using mixtures of different pigments, (in particular anthocyanins and betalains) to obtain more precise indications of food spoilage (in particular, hake spoilage).

## Figures and Tables

**Table 1:** Thickness, water solubility (WS), contact angle, wettability, mechanical properties, content of anthocyanins and betalains, and color parameters of the films.

Parameters	CS	CSBR	CSRC	CSBC
**Thickness (mm)**	0.36 ± 0.04 ^a^	0.47 ± 0.03 ^b^	0.43 ± 0.04 ^b^	0.42 ± 0.02 ^b^
**WS (%)**	29 ± 2 ^a^	36.6 ± 0.5 ^b^	30 ± 3 ^a^	37 ± 1 ^b^
**Contact angle (°) measured at** **20 s**	2.6 ± 0.7 ^a^	13.8 ± 0.8 ^b^	3.0 ± 0.6 ^a^	19.6 ± 0.5 ^c^
**Wettability (mN/m)**	−0.03 ± 0.04 ^a^	−2.1 ± 0.5 ^b^	−0.08 ± 0.09 ^a^	−4.2 ± 0.7 ^c^
**Stress at rupture (MPa)**	0.8 ± 0.1 ^ab^	0.62 ± 0.07 ^b^	1.2 ± 0.1 ^c^	1.0 ± 0.2 ^c^
**Deformation at rupture (mm/mm)**	2.7 ± 0.2 ^a^	1.2 ± 0.1 ^b^	0.8 ± 0.1 ^c^	0.9 ± 0.2 ^c^
**Betalains (mg/100 g)**	-	41.61 ± 0.08	-	8.9 ± 0.1
**Anthocyanin** **(mg C3G/100 g)**	-	-	37.9 ± 0.3	24.9 ± 0.4
**Color**				
L*	85.85 ± 0.04 ^a^	32 ± 1 ^b^	54 ± 1 ^c^	46 ± 2 ^d^
a*	−1.37 ± 0.01 ^a^	41 ± 2 ^b^	2.2 ± 0.6 ^a^	20 ± 1 ^c^
b*	5.41 ± 0.02 ^a^	23 ± 1 ^b^	2.6 ± 0.1 ^c^	15 ± 1 ^d^
∆E*	-	69 ± 1 ^a^	31.1 ± 0.4 ^b^	45 ± 1 ^c^

Mean and standard deviations for *n* = 3 are reported. Anthocyanin content is expressed as equivalents of cyanidin-3-glucoside (C3G). (-) no data. Same letter in a row means non-significant differences (*p* < 0.05) between data reported. CSBR: starch-BR film; CSRC: starch-RC film; CSBC: starch-BR/RC film.

**Table 2:** Microbial and physicochemical changes in fish stored at 4 °C.

Day	Systems-CSBR		Systems-CSRC		Systems-CSBC	
	Log CFU/g	TVB-N mg/100 g	Log CFU/g	TVB-N mg/100 g	Log CFU/g	TVB-N mg/100 g
**0**	3.16 ± 0.09 ^a^	5.6 ± 0.4 ^a^	3.16 ± 0.09 ^a^	5.6 ± 0.4 ^a^	3.16 ± 0.09 ^a^	5.6 ± 0.4 ^a^
**3**	6.1 ± 0.5 ^b^	10 ± 1 ^b^	6.4 ± 0.1 ^b^	12 ± 1 ^b^	6.0 ± 0.2 ^b^	12 ± 1 ^b^
**4**	6.9 ± 0.2 ^c^	31 ± 2 ^c^	^-^	-	6.7 ± 0.3 ^c^	26 ± 1 ^cd^
**5**	7.52 ± 0.07 ^d^	46 ± 3 ^e^	^-^	-	^-^	-
**8**	8.8 ± 0.1 ^e^	78.5 ± 0.7 ^f^	-	-	-	-

Mean and standard deviations for *n* = 3 are reported. (-) no data. For the same parameter, the same letter indicates non-significant differences (*p* < 0.05) between data reported.

## Data Availability

The data presented in this study are available on request from the corresponding author.
